# Trapping and Manipulation of Single Cells in Crowded Environments

**DOI:** 10.3389/fbioe.2020.00422

**Published:** 2020-05-08

**Authors:** Qian Zhao, Hao-Wei Wang, Pan-Pan Yu, Shu-He Zhang, Jin-Hua Zhou, Yin-Mei Li, Lei Gong

**Affiliations:** ^1^Department of Optics and Optical Engineering, University of Science and Technology of China, Hefei, China; ^2^Shandong Provincial Engineering and Technical Center of Light Manipulations and Shandong Provincial Key Laboratory of Optics and Photonic Device, School of Physics and Electronics, Shandong Normal University, Jinan, China; ^3^Department of Biomedical Engineering, Anhui Medical University, Hefei, China; ^4^Hefei National Laboratory for Physical Sciences at the Microscale, University of Science and Technology of China, Hefei, China

**Keywords:** optical tweezers, crowded environment, lymphocytes, optical shield, optical force

## Abstract

Optical tweezers provide a powerful tool to trap and manipulate living cells, which is expected to help people gain physiological insights at single-cell level. However, trapping and manipulating single cells under crowded environments, such as blood vessels and lymph nodes, is still a challenging task. To overcome this issue, an annular beam formed by the far-field Bessel beam is introduced to serve as an optical shield to isolate the target cells from being disturbed. With this scheme, we successfully trapped and manipulated single blood cells in a crowded environment. Furthermore, we demonstrated manipulation of two lymphocytes ejected from a lymph node independently with dual-trap optical tweezers, which paves the way for exploring cell interactions under living conditions. Such technique might be helpful in the study of how natural killer cells response to virus-infected cells or cancer cells.

## Introduction

Since the pioneer work of Ashkin et al. (1986), optical tweezers have emerged as an essential tool for manipulating single cells and performing biomechanical characterizations at microscopic level (Chen et al., 2007; Zhong et al., 2013b; Xie et al., 2018). Distinct advantages of using tweezers for these characterizations include non-contact cell manipulation, Piconewton force accuracy (Williams, 2002; Falleroni et al., 2018), and amiability to liquid medium environments (Zhang and Liu, 2008). The wide application range of optical tweezers, such as transporting foreign materials into single cells (Hou et al., 2016; Johansen et al., 2016), delivering cells to specific locations (Xie et al., 2018), sorting cells in microfluidic systems (Wang et al., 2011; Landenberger et al., 2012), and quantifying intercellular interactions (Chen et al., 2007; Hongliang et al., 2008), have been proved previously. However, researches readily performed *in vitro* may not reflect the biological activities *in vivo* accurately because of the complexity of the *in vivo* environment. Recently, trapping, and manipulation of cells within living animals has been achieved using infrared optical tweezers (Zhong et al., 2013a; Johansen et al., 2016). Notably, a non-contact micro-operation has been managed to clear a blocked capillary in a living mice (Zhong et al., 2013a). Therefore, the ability of manipulating single cells *in vivo* is urgently demanded to verify our knowledge acquired from the *in vitro* studies.

Optical tweezer is capable of probing the physical properties of cells in living animals with microscale resolution (Paul et al., 2019), which might help people gain physiological insights at single-cell level. A recent work (Johansen et al., 2016) performed a unique way to stimulate immune response by optically trapping and manipulating injected bacteria inside a living zebrafish. Although there is no unconquerable obstacle for optical trapping inside living animals, isolating single cells under crowded environments remains a challenging task. When optically manipulating *in vitro* (Xie et al., 2002; Sinjab et al., 2018), samples are usually diluted to sparsely populated level so that the individual cells can be manipulated, and measured without disturbances from the ambient ones. However, living environments are usually congested with various classes of cells, and ambient cells enter the trap consequently and frequently. Thus, the tracking procedure is disturbed and the accuracy of the measurement is reduced. For example, the crowded environment inside the lymph node (Stoll et al., 2002; Willard-Mack, 2006) might be the most important obstacle to optical tweezers studies in this critical small gland related to infection and cancer development. In brief, the crowded environments inside living animals become a critical challenge for manipulating single cells *in situ*.

Trapping with structured light beams is the frontier of optical tweezers. For example, the non-diffraction Bessel beam could overcome the limited depth of focus of Gaussian beam (Durnin et al., 1987). Bottle beams could help trap absorbing particles that cannot be stably confined by conventional optical tweezers (Gong et al., 2016). In this work, we trapped and manipulated single cells in the crowded environment with engineered trapping laser beam. Serving as an optical shield, an annular beam is introduced to avoid the disturbance from crowded environments during single cell manipulation. We adopt an optical configuration using a diffraction axicon and a converging lens to create the high efficiency annular beam. The established optical shield can be regulated by adjusting the distance between the axicon and the lens. Moreover, a theoretical analysis of the optical forces is presented. According to our analysis, the scattering force is stronger than the gradient force, and particles are propelled away from the center in the beam direction. As a proof of concept, we first trapped single blood cells within a crowded environment. Next, we applied the method to manipulate individual lymphocytes from a lymph node inside its normal physiological status. Our work is expected to benefit the research of the lymphocytes related immune response in the lymph nodes by keeping the natural physiological environment.

## Materials and Methods

### Principle of Generating Optical Shield

**FIGURE 1:**
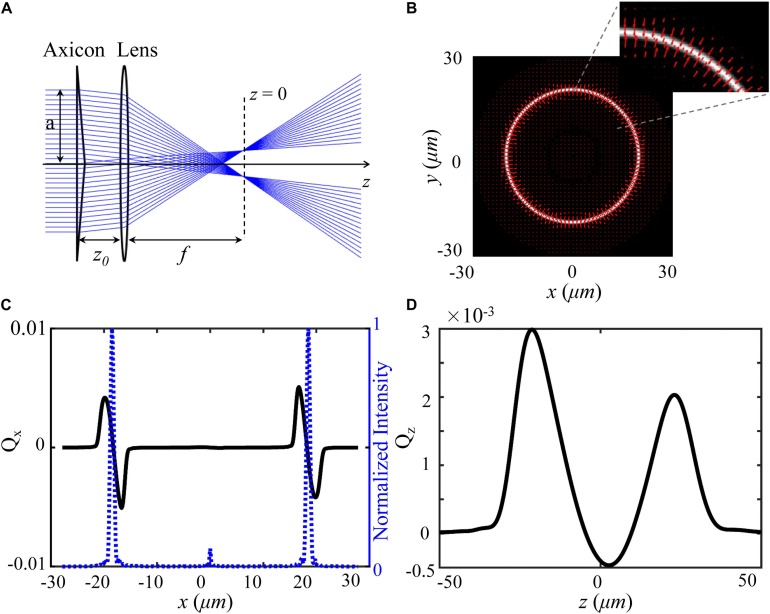
Model and force distribution of optical shield. **(A)** Experimental scheme for the adjustable optical shield. **(B)** The annular intensity distribution at the focal plane, where red arrows show the magnitude and direction of total force vectors as a function of the position. The inset presents an enlarged view of the force vectors. The force is calculated on a particle (radius = 2.5 μm, *n_*p*_* = 1.50) immersed in water (*n_*m*_* = 1.33). Transversal trapping efficiency *Q*_*x*_ **(C)** and longitudinal trapping efficiency *Q*_*z*_ **(D)** are plotted as a function of *x*-displacement and *z*-displacement, respectively.

To produce an optical shield, a hollow beam is constructed by an axicon and a lens as shown in Figure 1. When an aperture plane wave incidents on an axicon, a Bessel beam in the range *Z*_max_ = *a*/(*n*−1)γ can be created under the paraxial approximation, where γ is the angle between the conical surface and the flat surface, *a* is the semi-diameter of the aperture plane wave, *n* is the index of the axicon. According to the focusing properties of aperture Bessel beam (Wei et al., 2005), as presented in Figure 1A, the hollow beam can be produced behind the lens when *z_0_* < *f* < *Z*_max_, where *z*_0_ is the distance between axicon and lens, *f* is the focal length of the lens. We calculate the total force vector indicated along red arrows as a function of a particle’s position over *x*-*y* plane using ray optics model (Zhou et al., 2008; Shao et al., 2019). In the calculation, the particle (radius = 2.5 μm, *n_*p*_* = 1.50) is immersed in water (*n_*m*_* = 1.33), and other simulation parameters include λ = 1064 nm, *f* = 260λ, *z_0_* = *0.6f*, and γ = π/18 were applied also. Details of trapping efficiency calculation are described in Supplementary Material. A bright annular intensity distribution is tightly focused on the focal plane of the lens (*z* = 0), as shown in Figure 1B. The total force vectors indicated by the red arrows are presented in *x-y* plane as a function of particle’s position. The length and the direction of arrows represent the magnitude and direction of the total force, respectively. Figures 1C,D show the transversal trapping efficiency and longitudinal trapping efficiency for the particle as a function of particle’s displacement along *x* axis, respectively. The blue dash line indicates the corresponding one-dimensional normalized intensity distribution. The distribution of the force arrows and the two trapping efficiency curves illustrate us the manipulation capability of the annular beam. When the particle is initially located in the inner part of the annulus and subjected to a drag force outward in the radial direction, it will shift to the bright annular intensity where the transversal trapping efficiency and longitudinal trapping efficiency are balanced. Noted that the longitudinal optical force can be balanced by the gravity of the particle. Therefore, the hollow beam can clear a blank area in which the trapped individual cell can be shielded.

### Experimental Setup

**FIGURE 2:**
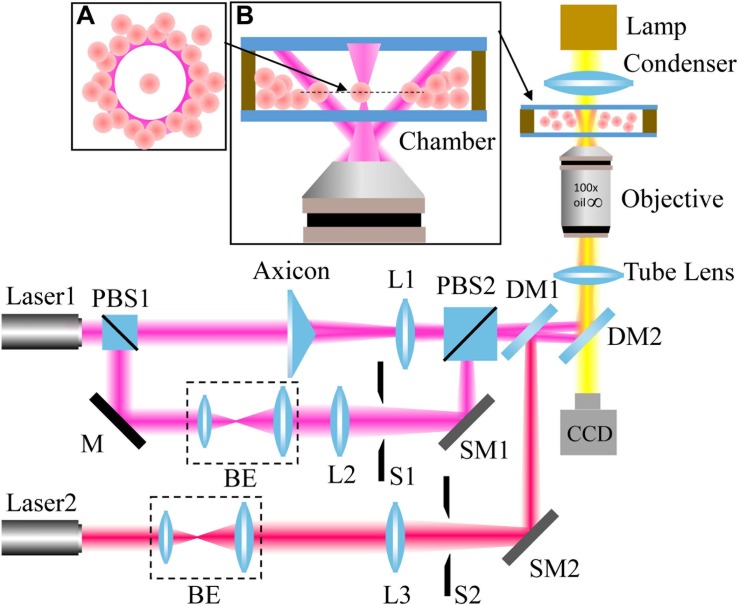
Experimental setup. L: lens; PBS: polarizer beam splitter; BE: beam expander; M: mirror; S: shutter; DM: dichroic mirror; and SM: scanning mirror. The wavelengths of Laser1 and Laser2 are 1064 nm and 780 nm, respectively. The insets: principle illustration of the optical shield **(A)** and magnified side view of the chamber **(B)**.

Our experimental setup is shown in Figure 2. A light beam from a 1064-nm continuous-wave (CW) laser was separated into two parts with a polarizer beam splitter (PBS1). The p-polarized beam passed through an axicon (γ = 0.5°, Thorlabs) to produce a Bessel-like beam. Then, the Bessel-like beam was focused by lens L1 to create an annular beam, which acts as an optical shield. The s-portion was expanded by a beam expander and finally focused by an objective lens to create an optical trap. In addition, a 780-nm CW laser serves as the light source for another optical trap. The three beam paths were combined together by PBS2 and a dichroic mirror (DM1). Another dichroic mirror (DM2) reflects the combined beams toward the back focal plane of the objective. After the beams propagate through a tube lens, a 100 × oil immersion objective lens (NA = 1.4, Olympus) highly focus them to create desired optical traps. To move the positions of the two focused optical spots, scanning mirrors based on the high-speed Piezo tip/tilt platforms (S-330.30, Physik Instrumente) were adopted to control the beam incidence. The state of optical tweezers can be switched “on” and “off” independently with shutters. As a result, an annular intensity distribution, and two controllable optical traps formed at the focal plane of the objective. The laser power used for 1064-nm light is higher than that of 780-nm light in order to provide enough power to push cells away. The laser powers of the optical traps are ∼120 mW (1064 nm), ∼120 mW (1064 nm), and ∼42 mW (780 nm). The whole optical tweezer setup was built on an Olympus IX71 inverted microscope, and real-time imaging of the trapping process can be readily realized with the microscope and a CCD camera.

### Sample Preparation

The samples used in the experiments are mice blood and an inguinal lymph node of mice. Mice were obtained from the Experimental Animal Center of University of Science and Technology of China. Mice blood was extracted from tail and diluted about 100 times with the isotonic Phosphate-buffered saline (PBS) buffer before injected into a glass chamber. Lymphocytes used in trapping experiment were ejected from the inguinal lymph node. An illustration cartoon (Supplementary Figure 1) was presented to illustrate procedure in detail. First of all, a lymph node was peeled off from mice and put into a chamber containing isotonic PBS. Then a cover glass was put on top of the lymph node to make it cling to the bottom of the chamber. Finally, we slightly pressed the cover glass so that the lymphocytes were ejected out of the lymph node via the efferent lymphatic. Since lymphocytes just left the lymph node, the biological activity and physiological status were well preserved (Miller et al., 2002; Stoll et al., 2002).

## Results

### Trapping a Single Blood Cell in a Crowd

**FIGURE 3:**
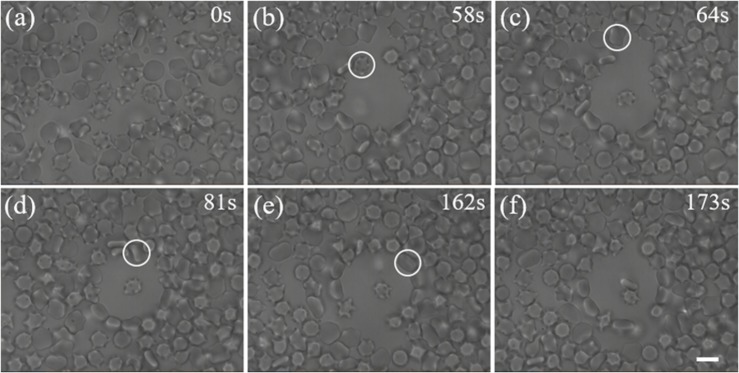
Trapping of individual cells in a crowded blood sample. **(a)** A crowd of blood cells before the optical shield is switched on. **(b)** A blank area was created by the optical shield. The labeled leukocyte is the first target we aim to capture. **(c)** The leukocyte was moved to the center of the shield with an optical trap. The labeled red blood cell is another target we try to capture. **(d, e)** The second cell was trapped and moved toward the first one with another optical trap. **(f)** Two trapped cells are manipulated to attach to each other. Scale bar: 5 μm.

We first tested the ability of our scheme for trapping single cells within a crowded environment using mice blood cells. Figure 3 shows the process of manipulating individual blood cells under crowded environment. At the very beginning (*t* = 0 s), there is a crowd of cells in the field of view. When the hollow beam was switched on, cells around the beam center were pushed outward to the high-intensity ring because of optical forces. After 58 s, an optical shield with many cells distributing around the circumference was established. As a result, there exists a blank area on the center of optical shield. Then, an additional optical trap was used to capture and manipulate a single blood cell. A leukocyte (highlighted by the white circle) was trapped and moved to the center of the blank area via active manipulation, in which the optical trap is moved by tilting the scanning mirror, as shown in Figure 3b at the moment of 64 s. At the same time, another red blood cell highlighted by the white circle in Figures 3c–e, was trapped and moved by another optical trap toward the first trapped cell (Figure 3e). Thus, two cells were isolated from the crowd and contacted with each other (Figure 3f).

### Manipulating Single Live Lymphocytes From a Lymph Node

**FIGURE 4:**
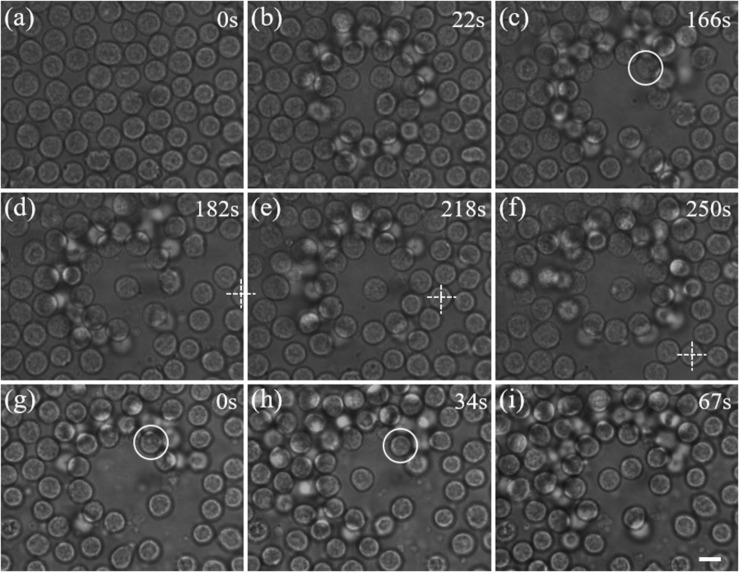
Manipulation of live lymphocytes ejected from a lymph node. **(a)** A dense distribution of lymphocytes. **(b)–(c)** A blank area is gradually created with the annular beam. **(c)–(d)** The cell in the white circle is trapped and moved to the center. **(d)–(f)** By moving the sample stage, the trapped cell can be moved in two dimension passively. The crossing indicates the reference point. **(g)–(i)** Independent manipulation of two lymphocytes in a crowd of lymphocytes. Scale bar: 5 μm.

We applied the proposed scheme for trapping individual lymphocytes from an inguinal lymph node. Direct optical trapping of lymphocytes inside the lymph node is difficult because the wide-field microscope is not practicable through the thick capsule of the lymph node. Therefore, the lymphocytes were first pushed out of the lymph node via the efferent lymphatic, and selected lymphocytes were trapped and isolated from a crowd in the area around the efferent lymphatic. This implementation mimics the environment inside the lymph node because the physiological status of lymphocytes did not change as they just left the lymph node. Figure 4a shows a picture of a crowd of lymphocytes. Firstly, we switched on the optical shield, which gradually pushed the cells out forward and created an empty area. Then a selected lymphocyte (highlighted by a white circle in Figure 4c) was trapped and moved to the center of the shield with a Gaussian trap, as shown in Figures 4b–d. The relative motion of optical tweezers is achieved by tilting the incident angle of the laser beam. Additionally, the target cell and the optical shield can be moved to a new position via stage motion, as illustrated in Figures 4d–f, where the white cross indicates the reference point.

Furthermore, we demonstrated manipulation of two lymphocytes ejected from a lymph node independently with dual-trap optical tweezers. As shown in Figures 4g–i, two lymphocytes highlighted by white circles are trapped and pulled together at the center of the blank area in sequence, which perfectly mimic exploring cell interactions at cellular level, and such as the responses of natural killer cells to virus-infected cells or cancer cells.

## Discussion

In conclusion, we have proposed and implemented an optical shield scheme for manipulating individual cells in a crowded environment. An adjustable optical shield was created by using a diffraction axicon and a converging lens. Optical forces exerted on micro-particles by the optical shield were calculated to analyze its capability for optical clearing. Trapping and manipulating individual living lymphocytes from a lymph node was achieved with our proposed scheme in experiment. Furthermore, we have demonstrated independent manipulation of two lymphocytes with dual-trap optical tweezers, which could benefit studies of cell interactions in a crowded environment.

Lymph nodes are critical small glands closely related to infection and cancer development for mammalians (Willard-Mack, 2006). Trapping and manipulation of individual cells inside the lymph nodes is expected to help the study of how natural killer cells response to virus-infected cells or cancer cells. In our current experiment, trapping lymphocytes was actually performed in the saline solution, and trapping depth through the lymph node capsule is limited to ∼100 μm due to the short working distance of the objective. A water immersion objective with longer working distance should be helpful to improve the trapping depth (Zhong et al., 2013b). However, the wavefront distortion caused by the thick capsule and limited imaging depth of the microscope are still obstacles of optical trapping inside the lymph node. Stripping the capsular surface of the lymph node or wavefront correction could be helpful in deep imaging and trapping. Because the inability of wide-field microscope in distinguishing T-cells and B-cells, fluorescence labeling technologies is demanded to recognize different kinds of cells, and which may promote the screening of immunological interactions (Stoll et al., 2002). In particular, our proposed trapping scheme can combined with some other techniques such as Raman spectroscopy (Sinjab et al., 2018), position tracking (McAlinden et al., 2014), and imaging (Berg and Holler, 2016) for advanced studies of individual live cells, where the optical shield will enable measurements without disturbances.

## Data Availability Statement

The raw data supporting the conclusions of this manuscript will be made available by the authors, without undue reservation, to any qualified researcher.

## Ethics Statement

The animal study was reviewed and approved by The Ethical Committee of the University of Science and Technology of China. Written informed consent was obtained from the owners for the participation of their animals in this study.

## Author Contributions

LG, QZ, and H-WW conceived the idea. QZ performed the experiment, analyzed the data. P-PY contributed to constructing the experimental setup. QZ, H-WW, and LG discussed the results. QZ and LG wrote and edited the manuscript. S-HZ and J-HZ contributed to the force calculation. Y-ML supervised the study. All the authors reviewed and revised the manuscript.

## Conflict of Interest

The authors declare that the research was conducted in the absence of any commercial or financial relationships that could be construed as a potential conflict of interest.
