# Supplementary material for: Trapping and Manipulation of Single Cells in Crowded Environments
Source: Front Bioeng Biotechnol. 2020 May 8;8:422. doi: 10.3389/fbioe.2020.00422 (PMC7227430; doi:10.3389/fbioe.2020.00422)
Supplement: Supplementary file 1 [file Table_1.DOCX]

Supplementary Material

# Supplementary note: calculation of trapping efficiency

1. Theoretical model of the annular beam created by an axicon

To numerically study the optical trapping performance, a model of the incident annular beam is required. For simplicity, the optical field of an aperture plane wave at the bottom of the axicon, which is located at $z=\left(-z_{0}-f\right)$, can be written as

$U_{0}\left[r,z=\left(-z_{0}-f\right)\right]=\left\{\begin{matrix} 1,\text{ }r\leq a \\ 0,\text{ }r>a \end{matrix}\right.$, (1)

where $r=\sqrt{x^{2}+y^{2}}$. The axicon can be regarded as a phase element. The output optical field is given by

$U\left[r,z=\left(-z_{0}-f\right)\right]=U_{0}\left[r,z=\left(-z_{0}-f\right)\right]\exp\left[-ik\sqrt{1-\left(1-n\right)^{2}\gamma^{2}}r\right]$. (2)

In order to calculate the optical field at the focusing plane (z=0), we propagate $U$ back to the front focal plane ($z=-2f$) using the Fresnel diffraction:

$\psi\left(r,z=-2f\right)=F^{-1}\left\{F\left(U\right)\exp\left[ik\left(z_{0}-f\right)\right]\exp\left[-i\pi\lambda\left(z_{0}-f\right)\left(f_{x}^{2}+f_{y}^{2}\right)\right]\right\}$, (3)

where $F$ denotes the 2D Fourier transform, and ($f_{x}$, $f_{y}$) are spatial frequencies. Then, the focused optical field can be approximately obtained by calculating its far field via Fourier transform:

$\Psi\left(\frac{x}{\lambda f},\frac{y}{\lambda f},z=0\right)=F\left(\psi\right)=F\left(U\right)\exp\left[ik\left(z_{0}-f\right)\right]\exp\left[-i\pi\frac{\left(z_{0}-f\right)}{\lambda f^{2}}\left(x^{2}+y^{2}\right)\right]$. (4)

1. Calculation of trapping efficiencies using Fourier ray method

The trapping efficiencies are calculated numerically by the Fourier ray method. According to the Fourier transform, the incident beam [Eq. (4)] is decomposed into a superposition of plane waves propagating in different directions. The plane waves are weighted by the angular spectrum [Eq. (3)]. Each plane wave can be regarded as infinite numbers of parallel rays (Fourier rays). The intensity of each Fourier ray is determined by the ratio between the total power of incident beam and the amplitude of corresponding angular spectrum. Then, the trapping efficiency for each Fourier ray is calculated according to the conventional ray optical method. Considering a microsphere with the refractive index of *n_p_* that is centered at $c\left(x_{c},y_{c},z_{c}\right)$. Let $M\left(x_{m},y_{m},z_{m}\right)$ be an arbitrary point on the microsphere, and its coordinates can be described by the parametric equations of the sphere:

$\left\{\begin{matrix} x_{m}=x_{c}+r\sin\eta\cos\xi\\ y_{m}=y_{c}+r\sin\eta\sin\xi\\ z_{m}=z_{c}+r\cos\eta\end{matrix}\right.$, (5)

where $r$ is the is the radius of the microsphere, and $\eta\in\left[0,\pi\right]$ and $\xi\in\left[0,2\pi\right]$. When a Fourier ray strikes on a tiny region of point M, the trapping efficiencies can be decomposed into a scattering component *Q_s_*:

$Q_{s}=1+R\cos\left(2\alpha\right)-T^{2}\left[\frac{\cos\left(2\alpha-2\beta\right)+R\cos\left(2\alpha\right)}{1+R^{2}+2R\cos\left(2\beta\right)}\right]$ , (6)

which is parallel to the incident ray, and a gradient component *Q_g_*:

$Q_{g}=-R\cos\left(2\alpha\right)+T^{2}\left[\frac{\sin\left(2\alpha-2\beta\right)+R\sin\left(2\alpha\right)}{1+R^{2}+2R\cos\left(2\beta\right)}\right]$, (7)

which is perpendicular to the incident ray. Parameters *α* and *β* are the incident angle and the refractive angle, which can be determined by spatial analytic geometry. *R* and *T* are the Fresnel reflection and transmission coefficients of energy flow. According to Eq. (3) and Eq. (4), the incident light power at point *M* of Fourier ray can be determined, yielding a finial expressions of the trapping efficiencies:

$\mathbf{Q}_{\chi}\left(x_{c},y_{c},z_{c}\right)\approx\iint_{x,y} \left[\iint_{\eta,\xi} Q_{\chi}I\left(x_{m},y_{m},z_{m}\right)\left|\cos\eta\right|\sin\eta\left|\psi\left(x,y\right)\right|^{2}\mathbf{v}_{\chi}\text{d}\eta\text{d}\xi\right]\text{ d}x\text{d}y,\chi=s,g$. (8)

Here, $\mathbf{v}_{\chi}$ is the directional vector for scattering and gradient optical force. $I\left(x_{m},y_{m},z_{m}\right)$ is the light intensity at point *M*, which can be calculated according to Eq. (4).

**
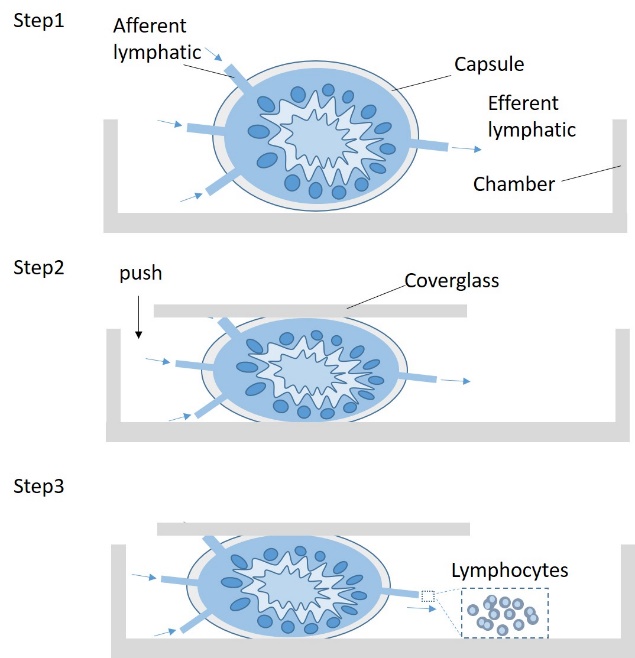
**

**Supplementary Figure 1.** The process of lymph node sample preparation. Step 1: The lymph node was put into a chamber containing isotonic phosphate buffered saline to maintain a normal osmotic pressure. Step 2: We put a cover glass on the lymph node to make it cling to the ground of the chamber. Step 3: We slightly pressed the cover plate and thus the lymphocytes were ejected out of the lymph node via the efferent lymphatic. The dashed box indicates the trapping area.
